# Enhanced Corneal Penetration of a Poorly Permeable Drug Using Bioadhesive Multiple Microemulsion Technology

**DOI:** 10.3390/pharmaceutics12080704

**Published:** 2020-07-26

**Authors:** Mohamed Moustafa Ibrahim, Doaa Nabih Maria, XiangDi Wang, Raven N. Simpson, T.J. Hollingsworth, Monica M. Jablonski

**Affiliations:** 1 Department of Ophthalmology, Hamilton Eye Institute, University of Tennessee Health Science Center, Memphis, TN 38163, USA; mibrahi2@uthsc.edu (M.M.I.); doaanabihmaria@gmail.com (D.N.M.); xwang17@uthsc.edu (X.W.); rdavi122@uthsc.edu (R.N.S.); thollin1@uthsc.edu (T.J.H.); 2 Department of Pharmaceutics, Faculty of Pharmacy, Mansoura University, Mansoura 35516, Egypt; 3 Department of Pharmaceutical Sciences, University of Tennessee Health Science Center, Memphis, TN 38163, USA

**Keywords:** microemulsion, corneal permeability, cytotoxicity, freeze–thaw, modified Draize test, ocular tolerance

## Abstract

Corneal penetration is a key rate limiting step in the bioavailability of topical ophthalmic formulations that incorporate poorly permeable drugs. Recent advances have greatly aided the ocular delivery of such drugs using colloidal drug delivery systems. Ribavirin, a poorly permeable BCS class-III drug, was incorporated in bioadhesive multiple W/O/W microemulsion (ME) to improve its corneal permeability. The drug-loaded ME was evaluated regarding its physical stability, droplet size, PDI, zeta potential, ultrastructure, viscosity, bioadhesion, in vitro release, transcorneal permeability, cytotoxicity, safety and ocular tolerance. Our ME possessed excellent physical stability, as it successfully passed several cycles of centrifugation and freeze–thaw tests. The formulation has a transparent appearance due to its tiny droplet size (10 nm). TEM confirmed ME droplet size and revealed its multilayered structure. In spite of the high aqueous solubility and the low permeability of ribavirin, this unique formulation was capable of sustaining its release for up to 24 h and improving its corneal permeability by 3-fold. The in vitro safety of our ME was proved by its high percentage cell viability, while its in vivo safety was confirmed by the absence of any sign of toxicity or irritation after either a single dose or 14 days of daily dosing. Our ME could serve as a vehicle for enhanced ocular delivery of drugs with different physicochemical properties, including those with low permeability.

## 1. Introduction

Unlike drug delivery to other parts of the body, ocular drug delivery has met with significant challenges due to the presence of multiple strict barriers that are inherent in ocular anatomy and physiology that help to protect this priceless organ from toxicants either from outside or inside the body [1]. Although it is not the only way, the corneal pathway is considered the major pathway for drug entry into the eyeball after topical drug application [2]. Drug penetration through the cornea may be hindered by several physiological and anatomical ocular barriers. Physiological or pre-corneal barriers include: formulation drainage; blinking; tear film; tear turn-over; and formulation-induced lacrimation. The anatomical barrier is related to the corneal structure [3]. A cornea exhibits a mechanical shield that greatly affects drug penetration due to two factors. The first factor is the tight junctions between juxtaposed corneal epithelial cells that resist movement of drug molecules between the adjacent corneal epithelial cells. The second factor is the diversity of corneal layer polarity. The epithelial layer is lipophilic in nature and hinders the penetration of hydrophilic drugs, whereas the stoma is hydrophilic, which prevents the penetration of lipophilic drugs. Therefore, to have the ability to pass through the cornea, the drug molecule should possess a certain degree of both hydrophilicity and lipophilicity (i.e., amphiphilic drug molecules). Collectively, both physiological and anatomical barriers result in a very small portion of the applied dose being absorbed and reaching its site of action inside the eyeball. Physiological barriers play their role by shortening drug–corneal contact time, and the anatomical barrier is the main cause of low drug corneal penetration [1,4]. There are two important criteria that should be available in a topically applied formulation that enhance ocular bioavailability. The first is the ability of the formulation to prolong its corneal contact time, and the second is its capability to enhance the corneal penetration of the drug molecules. Several formulation strategies have been used to prolong the drug–corneal contact time. Formerly, ointment bases were used as viscous vehicles for this purpose; however, their use is now limited due to the undesirable side-effect of blurred vision. The use of viscosity-inducing agents—cellulose derivatives [5] and hyaluronic acid [6]—to reduce the formulation drainage could replace the use of an ointment base. Additionally, the use of bioadhesive polymers, such as chitosan and its derivatives [7,8,9], sodium alginate, xanthan and carrageenan [10], may help to prolong the corneal contact time. 

Recently, corneal penetration enhancers have been widely used to improve drug permeability into the eye. Penetration enhancers are capable of causing a transient alteration in the corneal epithelial structure that results in an increase in the amount of drug that passes through the cornea either transcellularly (through the cell membrane) or paracellularly (through the spaces between cells) [11]. Although it causes a temporary alteration in the structure of the corneal epithelium, penetration enhancers should be safe and not cause any permanent damage and initiate a minimal irritant effect [12]. Examples of the commonly used penetration enhancers are chelators such as ethylenediaminetetraacetic acid (EDTA) [13], surfactants such as Brij 78 [14] and preservatives such as benzalkonium chloride [15]. Corneal permeability can also be enhanced using recent formulation strategies. One of the most widely employed formulation strategies in the field of ocular drug delivery is the colloidal drug delivery system; examples include nano and micro-particles [16,17], nanostructure lipid carriers [18], liposome, micellar formulation [19], polymeric micelles [20], nanosuspension, dendrimers and nano and micro-emulsions [1]. Most of these drug delivery systems are prepared from bioadhesive materials and contain one or more penetration enhancer in order to improve the corneal permeability of the incorporated drugs.

Corneal permeability can also be affected by the physicochemical properties of the drug itself. According to the Biopharmaceutical Classification System (BCS), a drug can be classified into four classes depending on the aqueous solubility and permeability through biological membranes. Class I drugs have high solubility and permeability. To enhance their efficacy, class I drugs should be formulated in bioadhesive formulations to improve corneal contact time and allow a sufficient amount to penetrate into the eye. Class II drugs have low solubility and high permeability. A formulation for this class of drugs should improve its solubility and corneal contact time. Class III drugs have high solubility and low permeability that require its formulation to improve its permeability and corneal contact time. Finally, class IV drugs have low solubility and permeability, which require a formulation to improve its solubility, permeability and corneal contact time [21].

We previously demonstrated the suitability of a multiple W/O/W microemulsion formulation for controlling the corneal permeability and sustaining the drug release of pregabalin, a model BCS class I drug. Although pregabalin has high water solubility and high permeability, we controlled its release for up to one day and its corneal penetration to adhere to a once daily dosing [22]. In the current study, we selected ribavirin, as a model BCS class III drug [23] that has high water solubility and low permeability. The novelty of our current work lies in challenging our formulation suitability for the improvement of the corneal penetration of a poorly permeable drug and to test the ability of our multiple microemulsion (ME) to sustain the in vitro release of such a highly-water-soluble drug. Ribavirin is a broad-spectrum guanosine analogue antiviral drug that produces activity against several RNA and DNA viruses [24]. It is commonly used in combination with interferon in the treatment of hepatitis C [25].

A multilayered W/O/W ME was employed due to its many advantages over other colloidal drug delivery systems, such as nanoparticles. These advantages include: ease of preparation; no need for high energy during its preparation; 100% encapsulation efficiency, as there is no drug loss during its preparation; higher physical stability upon storage; and the presence of surfactants, which act as penetration enhancers that improve the permeability of the incorporated drug. Compared to other types of microemulsions (O/W and W/O), a multilayered W/O/W ME has the combined advantages of both types of microemulsions, while avoiding their individual drawbacks. Regarding the advantages, a W/O/W takes advantage of the slow release behavior of the W/O emulsion type, and the aqueous sensation of the O/W emulsion type. In addition, it avoids the greasy effect of the W/O emulsion type, and the rapid release behavior of the O/W emulsion type.

In the current study, we used ribavirin as a model drug. We strove to demonstrate the suitability of our multiple W/O/W ME formulation to sustain the drug release and improve the corneal permeability of this model BCS class III drug.

## 2. Methods and Materials

### 2.1. Materials

Ribavirin was supplied as a gift from Sigma Pharmaceutical Industries (Monufia, Egypt); sodium phosphate dibasic, sodium bicarbonate, potassium chloride, potassium dihydrogen phosphate, calcium chloride dihydrate, dipotassium hydrogen phosphate, Triton X-100, Cremophore EL, methyl thiazol tetrazolium (MTT) and glutathione disulfide were purchased from Sigma-Aldrich (St. Louis, MO, USA). Propylene glycol, magnesium chloride hexahydrate, dextrose, sodium chloride, gastric mucin (type II), dimethyl sulfoxide (DMSO), phosphoric acid and methanol (HPLC grade) were purchased from Fisher Scientific (Fair Lawn, NJ, USA). Gift samples of Labrafac Lipophile WL1349, Capryol 90 and Labrasol were obtained from Gattefossé Corporation (Paramus, NJ, USA). Soybean L-α-lecithin (97.7% phosphotidyl choline) was purchased from Calbiochem (Billerica, MA, USA). Ethyl alcohol was purchased from Decon Laboratories, Inc. (King of Prussia, PA, USA). Carbopol 981 was obtained as a gift sample from Lubrizol Advanced Materials, Inc. (Cleveland, OH, USA). Sodium alginate (viscosity of 1% solution at 25 °C = 5−40 cP) was purchased from MP Biomedicals (Solon, OH, USA). The immortalized human corneal-limbal epithelial cell line (HCLE) was supplied by Dr. Ilene K. Gipson (Harvard Medical School) [26]. Keratinocyte SFM serum free medium was purchased from Life Technologies Corporation (Grand Island, NY, USA). DMEM/F-12 (Dulbecco’s modified Eagle’s medium/nutrient mixture F-12) was purchased from Mediatech, Inc. (Manassas, VA, USA). Fresh whole eyes of male New Zealand white rabbits were obtained from Pel-Freez Biologicals (Rogers, AR, USA).

### 2.2. Animals

Dutch belted rabbits (*n* = 6, equally balanced between males and females, weighing 1.5−2.0 kg) purchased from Covance Inc. (Princeton, NJ, USA) were used to test the ocular tolerance of our formulations. All procedures including rabbits were approved by the Animal Care and Use review board of the University of Tennessee Health Science Center (UTHSC) (protocol number 18-059, approval date August 2018) and followed the Association of Research in Vision and Ophthalmology (ARVO) Statement for the Use of Animals in Ophthalmic and Vision Research in addition to the guidelines for laboratory animal experiments (Institute of Laboratory Animal Resources, Public Health Service Policy on Humane Care and Use of Laboratory Animals).

### 2.3. Methods

#### 2.3.1. Ribavirin HPLC Assay

Ribavirin was quantified in all in vitro experiments using the previously published reversed phase HPLC method [27]. Briefly, an Agilent 1100 series HPLC system (Waldbronn, Karlsruhe, Baden-Württemberg, Germany) was attached to Supelco kromasil C18 column (5 μm, 100 Å, 4.0 mm Å~300 mm). The mobile phase consisted of a mixture of 95% of 0.01M potassium dihydrogen orthophosphate and 5% methanol (pH 4.6) at a flow rate of 1 mL/min. The effluent was monitored by photodiode array detector and ribavirin was detected at 207 nm. 

#### 2.3.2. Preparation of Bioadhesive Multiple W/O/W Microemulsion 

The bioadhesive multiple W/O/W ME was prepared in 3 steps according to our recently published protocol [22]. Briefly, the ratios of the primary ME were selected from a previously constructed pseudo-ternary phase diagram. The selected ratios of the primary phase were 50% water, 10% labrafac lipophile WL1349 and 40% hydrophobic surfactants mixture (soybean lecithin and capryol 90, 1:1). Ribavirin was dissolved in the water of the primary ME, which in turn was emulsified by simple vortex shaking in the intermediate oil phase with the aid of hydrophobic surfactants mixture to produce the primary W/O ME. The primary W/O ME was then re-emulsified by simple vortex shaking in the external aqueous phase, which consisted of 70% water, 20% propylene glycol, 5% labrasol and 5% cremophor EL, in which the bioadhesive polymer (sodium alginate or Carbopol 981) was previously soaked. The ratio of the primary W/O ME to the external aqueous phase was 1.1:10. The ME components and their ratios were determined through conducting preliminary studies that included the screening of different surfactants and construction of several ternary and pseudo-ternary phase diagrams. The percentages of the components of the final ribavirin-loaded ME were listed in Table 1. 

#### 2.3.3. Microemulsion Physical Stability Tests

(a) Freeze–Thaw Cycles Test

Three different batches of drug-loaded Carbopol 981 ME were prepared and subjected to three alternative freeze (at −20 °C) and thaw (at 25 °C) cycles. The duration of each cycle was 48 h. At the end of each cycle, the formulation was assessed for its physical appearance, regarding phase separation, precipitation and any appearance of turbidity or cloudiness [28].

(b) Centrifugation Test

Three separate batches of the drug-loaded Carbopol 981 ME were subjected to centrifugation (SORVALL, WX Ultra Series Centrifuge, Newtown, CT, USA) at 25 °C to assess the physical stability against different centrifugation forces: 5000, 10,000, 20,000, 40,000, 50,000 and 60,000 rpm. The ME was centrifuged for 30 min at each centrifugation speed then assessed for its physical appearance after each cycle [29]. 

#### 2.3.4. Microemulsion In Vitro Evaluations

(a) Droplet Size, Polydispersity Index (PDI) and Zeta Potential Measurements

The ME droplet size, PDI and zeta potential were measured using a zetasizer (Nanoseries, nano-ZS, Malvern Instruments Limited, Cambridge, UK) after suitable dilution. Each experiment was repeated three times and the results were presented as means ± SEMs.

(b) Transmission Electron Microscopy Examination

The droplet distribution and the droplet morphology of ribavirin-loaded Carbopol 981 ME were examined using transmission electron microscope (TEM) (JEOL JEM1200EX II, Peabody, MA, USA) according to the following protocol: The ME was diluted 1:100 with distilled water. Two microliters of the diluted ME was placed on 400 mesh copper grids covered with ultra-thin Formvar film (Electron Microscopy Sciences EMS, Hatfield, PA, USA). The grids were incubated in a desiccator and allowed to dry. The dried grids were negatively stained with Uranyless EM stain (Electron Microscopy Sciences EMS, Hatfield, PA, USA) before examination by TEM.

(c) Microemulsion Viscosity Determination

The viscosity of ribavirin-loaded ME was measured using a cone and plate rotary viscometer (Brookfield DV-II+; Brookfield Engineering Laboratories, Middleboro, MA, USA) at 35 °C according to our previously published protocols [22,30,31]. Briefly, 0.5 mL of the formulation was placed between the cone and plate and allowed to equilibrate with the machine temperature for 5 min before measuring. Measurements were done in triplicate and the results were plotted as mean ± SEM.

(d) Measuring Microemulsion Bioadhesive Force

The bioadhesive force of our ME was determined using the viscosity method according to previously published protocols [32,33]. This method depended mainly on measuring the change that happened in the formulation viscosity upon mixing with mucin dispersion, which was then translated to bioadhesion force using the following equations: 

η_b_ = η_mix_ − (η_m_ + η_f_) (1)

F_b_ = η_b_ × Y (2)

Briefly, the viscosities of the ME formulation (η_f_), 15% *w*/*v* mucin type II dispersion in artificial tears fluid (η_m_) and the formulation/mucin mixture (η_mix_) were measured using cone and plate rotary viscometer at 35 °C. Viscosity improvement due to bioadhesion (η_b_) was calculated from Equation (1) and the bioadhesion force was calculated from Equation (2) by multiplying (η_b_) by the shear rate (Y).

(e) In Vitro Drug Release

To test the ability of our ME to sustain drug release, ribavirin release from MEs or the control formulations was studied using two compartment fast equilibrium microdialyzers (Harvard Apparatus Co., Holliston, MA, USA) in which a regenerated cellulose membrane (1000 Da molecular weight cut off) was mounted between the two chambers. The donor chamber contained 100 mg of the ME or the control formulation, while the receiver chamber was filled with 1.5 mL of phosphate buffered saline (PBS), pH 7.4. The control formulations were ribavirin solutions in water, sodium alginate or Carbopol 981 polymeric solutions at the same concentration used in the MEs. The dialyzers were shaken at 50 rpm in a thermostatically controlled shaker that maintained at 35 °C. The entire medium in the receiver chambers was removed and replaced by a fresh warm PBS (at 35 °C) at predetermined time intervals ranging between 0.25 and 24 h. All withdrawn samples were immediately assayed for their drug contents using the previously mentioned HPLC method [22,34]. The cumulative amount released was calculated as a percentage from the total drug content of the 100 mg of the same formulation batch. Each experiment was repeated three times and the results were plotted as means ± SEMs. 

For a precise calculation of the percentage of the cumulative amount released, the 100% drug content of each formulation was determined as follows: 100 mg of each formulation was diluted in 10 mL of water/ethanol mixture (3:7) for ME and water only for other formulations. Diluted samples were shaken for 10 min and filtered through 0.22µm membrane filter. The clear filtrates were directly assayed without further dilution for their drug contents by the HPLC method described before. Each experiment was performed thrice and the data were represented as means ± SEMs. The obtained actual drug contents were used in calculation of the percentage cumulative amount released.

(f) In Vitro Transcorneal Permeability Study

The ability of our ME to improve the corneal penetration of ribavirin, a poorly permeable drug, was tested by measuring the corneal permeability of our formulations using freshly separated corneas from whole New Zealand rabbit eyes obtained from Pel-Freez Biologicals (Rogers, AR) and shipped overnight in Hanks balanced salt solution over wet ice. The corneas were mounted on modified rounded junction Franz diffusion cells (PermeGear Inc., Hellertown, PA, USA) with the epithelial side upright toward the donor chamber and the endothelial side facing down toward the receiver chamber. The formulations were placed in the donor chamber directly on the corneal epithelial side. The receiver chamber was filled with 5 mL BSS-Plus solution (Alcon Laboratories Inc., Fort Worth, TX, USA) which was continuously stirred by a magnetic stirrer through the whole experiment. The temperature of the Franz diffusion cells was maintained at 35 °C by a circulating water bath; 500 μL of each of the solutions in the receiver chambers was withdrawn at predetermined time intervals ranging from 1 to 6 h and immediately replenished by a fresh, warm BSS-Plus solution. The withdrawn samples were assayed for their drug contents using the HPLC method mentioned previously. Each formulation was evaluated in six replicates and the cumulative amount permeated was calculated as mean ± SEM. The steady state flux (J) and the permeability coefficient (P) were calculated from the following equations [22,35]:

J = (dM/dt)/A(3)

P = J/Cd(4)

where: dM/dt is the cumulative amount (M) permeated per unit of time (t) (i.e., permeation rate); A is the surface area of the cornea through which drug permeation occurs (i.e., 0.636 cm^2^); and Cd is the initial drug concentration in the donor chamber. The relative improvement in the transcorneal permeation was calculated as a ratio of the permeability coefficient of ribavirin in the formulation to that in the aqueous solution.

(g) In Vitro Evaluation of ME Effect on Cell Viability

The potential cell toxicity of our ME was evaluated in vitro using methyl thiazolyl tetrazolium (MTT) assay according to our previously published protocols [22,36,37]. Because our formulations are intended to be used topically in the eye, a human corneal limbal epithelial cell line (HCLE) was used to evaluate the effect that our ME has on epithelial cell viability [26]. Briefly, a 96-well plate (Costar 3596; Corning Inc., Corning, NY, USA) was seeded by HCLE cells (14,000 cell/well) in 200 µL Dulbecco’s modified Eagle’s medium and kept overnight in a humidified atmosphere in incubator at (37 °C and 5% CO_2_) in order to allow for cell attachment to the well bottom. The next day, medium was withdrawn and replaced by 50 µL contrived tears fluid (Ursa Bioscience LLC, Bel Air, MD, USA) and 150 µL of the diluted formulations. The ME and the control formulations were diluted by Dulbecco’s modified Eagle’s medium in a ratio of 2.15 µL formulation to 147.85 µL medium. The formulation dilution was based on the relative surface area of the cell layer in the well of the 96-well plate exposed to the formulation compared to the real ocular surface area of the human or the rabbit exposed to the eye drop. Cells were incubated with the formulations for two different time intervals, 1 h and 2 h. At the end of the incubation time, the formulations were removed; cells were washed twice by medium; and then 200 µL of MTT solution (1mg/mL) was added to each well. After 4 h in the incubator, MTT solution was replaced by 200 µL DMSO, and the whole plate was shaken for 15 min to dissolve the formazan crystals. The optical absorbance of the formazan solution was measured at 570 nm using a plate reader (μ-Quant, Bio-Tek Instruments, Inc. Winooski, VT, USA). The absorbance was translated to a percentage of cell viability by dividing the optical absorbance of the formulation by that of the negative control (untreated cells, incubated in medium only) × 100. Each experiment was repeated eight times and the results were expressed as means ± SEMs. 

#### 2.3.5. In Vivo Safety and Ocular Tolerance Evaluation

(a) Acute Ocular Toxicity Evaluation (Modified Draize Test)

Some ophthalmic formulations may cause ocular irritation or toxicity after a single topical application. Although our ME formulations were synthesized using biocompatible ingredients, we tested them for possible acute ocular toxicity or irritation upon a single topical ocular application. In the experiment, we used ribavirin-loaded Carbopol 981 ME as the medicated formulation and drug-free Carbopol 981 ME as the blank formulation. Six Dutch belted (DB) rabbits (3 males and 3 females) were used for this study. Each rabbit was placed in a 4 kg rabbit restrainer (Plas Labs Inc., Lansing, MI, USA) to prevent the rabbits from rubbing their eyes during the first 4 h of the experiment. All rabbit eyes were examined by the naked eye for any problems or abnormalities before the test. Each rabbit received a single dose of 100 µL of the drug-loaded ME in the lower conjunctival sac of one eye; meanwhile the contralateral eye received a single dose of 100 µL of the blank ME. All eyes were observed by the naked eye for any signs of irritation, toxicity or allergic reactions—tearing, inflammation, conjunctival redness, conjunctival swelling, corneal swelling, etc., after 1, 2, 3, 4, 6, 8, 24, 48 and 72 h from the application.

(b) Chronic Ocular Toxicity Evaluation

To confirm the safety and biocompatibility of our ME formulation after prolonged use (14 days), the ME was tested in DB rabbit eyes according to the following protocol. Six DB rabbits, balanced between males and females, were subjected to the study. Each rabbit received one daily dose of 100 µL of ribavirin-loaded Carbopol 981 ME (medicated) in the lower conjunctival sac of one eye; meanwhile, the contralateral eye received 100 µL of drug free Carbopol 981 ME (blank) for 14 consecutive days. Rabbits’ eyes were evaluated daily by the naked eye for the occurrence of any signs of toxicity or allergic reactions, such as tearing, redness, swelling, inflammation or corneal abrasion. In addition to the daily evaluations, all rabbits’ eyes were subjected to fundus exam and slit-lamp biomicroscopic examinations before starting dosing and after 24 h of the last dose using both narrow and wide beam exams. The narrow beam exam was used to detect any complications that may arise in the cornea or in the anterior chamber, such as corneal inflammation, corneal swelling, change in the corneal surface smoothness or a change in the aqueous humor clarity. The purpose of the wide beam exam was to examine the whole eye surface and detect any changes or inflammation in conjunctiva, cornea or the eyelid. 

#### 2.3.6. Statistical Analysis

Data were statistically analyzed using one-way analysis of variance (ANOVA) test followed by Tukey’s multiple comparisons post-hoc test or an unpaired *t*-test. Statistical calculations were carried out using GraphPad Prism 8 software (GraphPad Software Inc., San Diego, CA, USA).

## 3. Results and Discussion 

We previously confirmed the suitability of our ME to sustain the drug release, control the corneal penetration and extend the pharmacological effect of pregabalin, a BCS class I drug. Although it has high solubility and permeability, our formulation succeeded in sustaining its corneal permeability and prolonging its corneal contact time [22]. 

Ribavirin, a BCS class III drug with high solubility and poor permeability, is mainly actively-absorbed after oral administration from the proximal small intestine through the gastrointestinal sodium-dependent nucleoside transporters [38]. The lack of passive absorption pathway, due to its high hydrophilicity, resulted in only 40% of the administered dose reaching the systemic circulation [39]. In the current study, we selected ribavirin as a model drug with different physicochemical properties than pregabalin (BCS class I) to challenge the ability of our ME to successfully deliver drugs from different BCS classes to the eye. Ribavirin has a high aqueous solubility that may result in a high release rate with poor corneal permeability. Therefore, here we strived to use our ME to improve the physicochemical properties of ribavirin by increasing its corneal penetration and sustaining its release rate. The novelty of the current work lies in the ability of our ME to improve the corneal permeability of a poorly permeable drug. 

### 3.1. Preparation of the Bioadhesive Multiple W/O/W Microemulsion 

To produce a stable ME, we selected the pseudo-ternary phase diagram with the largest microemulsion area from the constructed diagrams for the primary W/O ME. Because ribavirin is a highly-water-soluble compound and its recommended dose is only 0.1% (according to the commercially available antiviral ribavirin eye drops, RibaCare^TM^, 8mg/8mL, AdvaCare Pharma USA, Cheyenne, WY, USA), only a small volume of water was needed to dissolve it. In addition, the high water solubility of the drug helped to incorporate the required dose in a small volume of the primary W/O ME (9.9% of the final W/O/W ME), which required a smaller ratio of hydrophilic surfactants (4.5% Labrasol + 4.5% Cremophor EL) in the external aqueous phase to emulsify them and produce a stable final W/O/W ME. The production of an ME with a lower surfactant content is a very important issue regarding the safety of our formulation because the reduced surfactant content is likely to cause less irritation. 

The ingredients of our ME were carefully selected to produce safe, biocompatible, chemically and physically stable products. The oil phase consisted of Labrafac Lipophile WL1349, which is a medium chain triglyceride ester of a saturated fatty acid that decreases the possibility of rancidity (i.e., oil oxidation) upon storage due to the absence of unsaturated bonds, which act as free-radical-attacking centers. The presence of such oil helps to improve the stability and prolong the shelf life of the ME [40]. Additionally, being a medium chain triglyceride makes it more stable against oxidation and provides it a higher solvent capacity than long chain triglycerides [41]. The lipophilic surfactants mixture that is responsible for the formation of the primary W/O ME consists of Capryol 90 and soybean lecithin. Capryol 90 is a propylene glycol ester of a medium chain fatty acid (caprylic acid), which has a similar chemical composition to the oil phase; that resulted in perfect compatibility between them. Soybean lecithin is a highly biocompatible surfactant due to its phospholipid nature that resembles the composition of the biological membranes [42]. The external aqueous phase of our ME consists of a hydrophilic surfactant mixture (Labrasol and Cremophor EL, 1:1), a cosurfactant (propylene glycol) and water in which the bioadhesive polymer (sodium alginate or Carbopol 981) is soaked. It is reported in the literature that Labrasol may cause mild eye irritation at a concentration of 5% and the severity of the irritation is proportional to its concentration [43]. For this reason, we used it at a concentration of 4.5% to be safe and to not cause any irritant effect on the eye. Similarly, Cremophor EL is used in a concentration of 4.5% which is greatly below the concentration that may cause eye irritation, as it has been reported to be safe to the eye up to a concentration of 30% [44,45]. In addition to being safe up to 50% concentration [46], propylene glycol has two important roles in our ME. The first role is as a cosurfactant to improve the physical stability and prevent phase separation of the ME. The second role is to add a demulcent effect to our ME in order to relieve any irritant effect that may result from the presence of surfactants [47]. 

Sodium alginate and Carbopol 981, were selected as bioadhesive polymers to instill bioadhesiveness to our ME due to their safety and biocompatibility. The safety of sodium alginate has been confirmed in multiple studies and it is extensively used as a safe and biocompatible vehicle for ophthalmic drug delivery systems [48,49,50]. Carbopol 981 is a non-benzene Carbopol, which is one of the safest Carbopol polymers due to the absence of benzene that may remain as solvent residue during its manufacturing [51]. Because of this, it is considered as one of the most suitable polymers for ophthalmic use. All the previously-mentioned advantages of the ME ingredients indicated that we carefully selected its components in order to produce a safe and biocompatible final product. 

### 3.2. Microemulsion Physical Stability

The purpose of the ME stability tests, freeze–thaw cycle and centrifugation tests, was to test the ME stability against different temperatures and mechanical stresses that may face it during shipment, transport and/or storage. Our ME exhibited excellent stability against both tests. During the freeze–thaw test, our ME showed cloudiness upon storage at −20 °C, which rapidly (1–2 min) disappeared upon shifting to 25 °C. Upon completing the three freeze–thaw cycles the ME still kept its original clear appearance and texture without any observed change. The main factor that was responsible for the rapid disappearance of the cloudiness upon removal from the freezer and the high stability of the ME against temperature changes was the presence of propylene glycol. It is known that propylene glycol freezes at much lower temperature than water and it is commonly used as antifreeze for cars. Additionally, it is commonly used as cryoprotectant for tissues [52] and to protect the consistency and mitigate the agglomeration of vaccines induced by the freeze–thaw effect [53]. Regarding the centrifugation test, all ME batches were physically stable because they did not show phase separation, creaming, cracking, precipitation, cloudiness or turbidity.

### 3.3. Microemulsion In Vitro Evaluations

#### 3.3.1. Droplet Size, Polydispersity Index (PDI) and Zeta Potential Measurements

The droplet size, PDI and zeta potential of both blank and medicated MEs were listed in Table 2. The droplet size of both sodium alginate and Carbopol 981 Mes, either blank or medicated, is approximately 10 nm, which is responsible for the clear and transparent appearance of our ME. Additionally the PDI, which reflects the droplet size distribution, has a small value, especially for Carbopol 981 ME, which indicates that the ME droplets have a uniform droplet size. This small uniform droplet size may have resulted from the presence of propylene glycol as a cosurfactant. The cosurfactant, upon mixing with the surfactant mixture, helped to decrease the viscosity of the surfactants film at the interface between the primary W/O ME and the external aqueous phase that resulted in more efficient emulsification and the breakdown of the W/O droplet into smaller ones and production of ME droplets with smaller diameters [44,54]. This tiny and uniform droplet size is a very important factor for achieving high corneal penetration, especially for such a poorly permeable drug (ribavirin), which requires a carrier such as a ME to increase its penetration. As previously mentioned, ribavirin can only be absorbed from the small intestine through an active transport system and cannot pass biological membranes by a passive process [38]. Unfortunately, this transporter is only available in the proximal small intestine. ME, as a colloidal carrier, is capable of changing this characteristic of ribavirin, allowing it to pass through biological membranes using a passive process due to its tiny droplet size. Seijo et al. and Scholes et al. stated that colloidal systems with particle sizes below 200 nm could easily pass through biological membranes via passive transport while carrying their drug contents across the biological membranes [55,56]. Our MEs possess negative zeta potentials due to the negative nature of the used polymers as a result of the presence of exposed free carboxylic acid groups on their surface. The value of zeta potential of our prepared MEs is approximately −20 mV, which is enough to ensure a good shelf life stability due to the repulsion between the ME droplets that prevent their coalescence upon storage.

#### 3.3.2. Transmission Electron Microscopy Examination

**Figure 1:**
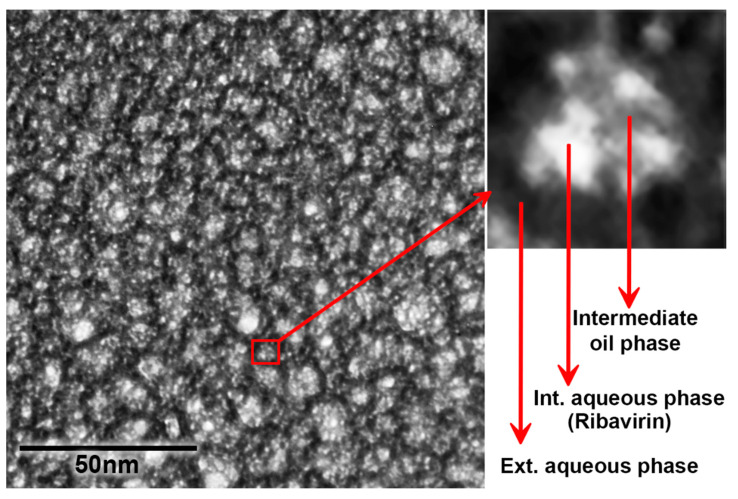
TEM examination of the diluted Carbopol 981 ME negatively stained with Uranyless illustrates the spherical shape and the multilayered structure of the ME globules. The magnified globules clearly show the presence of several water droplets inside the oil droplet, which is surrounded by the continuous external aqueous phase.

Transmission electron microscopy examination showed the spherical shape of the ME globules and confirmed the tiny size of the droplets (≈10 nm) that were obtained from a zetasizer. The three-layered structure of ME is clearly seen in the figure of the magnified globule with several droplets of the internal aqueous phase that are distributed inside a droplet of the intermediate oil phase, which is surrounded by the continuous external aqueous phase (Figure 1).

#### 3.3.3. Microemulsion Viscosity Determination

**Figure 2:**
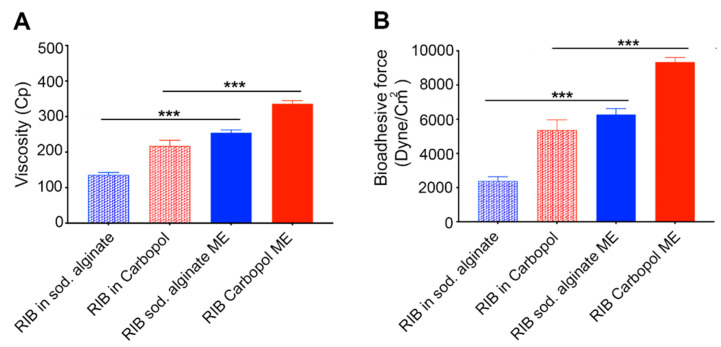
(**A**,**B**). Viscosity and bioadhesive force values of ribavirin-loaded Carbopol 981 and sodium alginate MEs and polymeric solutions, respectively. ME formulations possess higher viscosity and bioadhesive force than the polymeric solution formulation, regardless of the type of polymer, and Carbopol 981 formulations have higher viscosities and bioadhesive forces than those of sodium alginate. *N* = 3, *** = *p* < 0.001.

Figure 2A, illustrates the viscosity values of ribavirin-loaded MEs (Carbopol 981 ME and sodium alginate ME) and their control polymeric solutions (Carbopol 981 and sodium alginate). It is evident that the ME greatly enhances the viscosity of the formulation compared to their control polymeric solutions for both Carbopol 981 and sodium alginate formulations (*p* < 0.001). The formulation viscosity is a very important factor, especially for topically-applied ophthalmic formulations. The viscosity of our formulation allowed it to be easily dispensed from the dropper container. In addition, it is viscous enough to remain inside the eye upon application without rapid drainage for enough time to allow the bioadhesive reaction to occur. Comparing the viscosity values of Carbopol 981 and sodium alginate formulations proved that Carbopol 981 formulations of either ME or polymeric solution have higher viscosities than the corresponding sodium alginate formulations (*p* < 0.01). This difference in the viscosity values may be due to the inherent properties of each polymer, such as molecular weight, crosslinking and concentration [57].

#### 3.3.4. Measuring Microemulsion Bioadhesive Force

The bioadhesive force values of our MEs and their control polymeric solutions are demonstrated in Figure 2B. It is obvious that the MEs possess higher bioadhesion force values than their corresponding polymeric solutions (*p* < 0.001). The increased bioadhesive force of the ME may be due to two factors. The first factor is the presence of the non-ionic surfactants that could improve the polymers chains’ wetting and swelling which in turn would result in better interaction with mucin, and so, better bioadhesion [58]. The second factor that may improve the bioadhesive properties of the ME is the increased surface area offered by the emulsification process which could result in a better interaction with mucin and hence improved bioadhesion [59]. Figure 2B also illustrates that both Carbopol 981 formulations (ME and polymeric solution) have higher bioadhesive forces than those of sodium alginate (*p* < 0.01). This may be due to the crosslinked structure of Carbopol 981 compared to the linear structure of sodium alginate [60]. This crosslinking structure provides Carbopol 981 with more elasticity and flexibility that results in a better interaction with mucin and high bioadhesion force [58]. 

#### 3.3.5. In Vitro Drug Release

**Figure 3:**
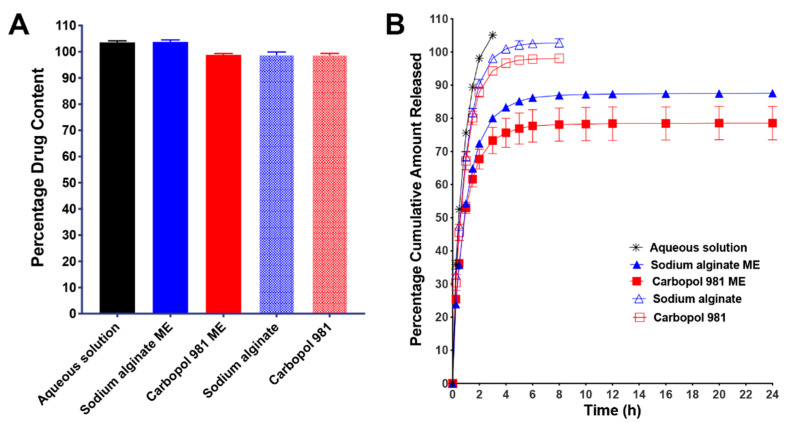
(**A**) Percentage drug content of ME and control formulations. (**B**) In vitro drug release profiles of ribavirin from ME and control formulations. The figure showed that the MEs possessed an initial burst release in the first few hours followed by sustained release behavior for up to 24 h compared to the control formulations that had fast drug release within few hours.

Figure 3A plots the percentage drug content of each ME and control formulation. These data demonstrate that all formulations possess a uniform drug content that ranged between 98.9% and 103.8%. The actual values of the drug contents were used as 100% in calculation of the percentage cumulative amount released.

The drug release profiles of ribavirin from ME (Carbopol 981 ME and sodium alginate ME) and the control (aqueous solution, Carbopol 981 polymeric solution and sodium alginate polymeric solution) formulations are illustrated in Figure 3B. It is evident that our MEs succeeded in sustaining the drug release compared to the control formulations. Control formulations exhibited 100% drug release within few hours (3 h for aqueous solution and 8h for polymeric solutions), while after 24 h of continuous release, our MEs did not reach 100% drug release. The fast drug release from an aqueous solution may be due to the high solubility of ribavirin in water and its availability in direct contact with the diffusion membrane (regenerated cellulose membrane) with the continuous removal of the release medium from the receiver chamber of the dialyzer, which ensures sink conditions. For polymeric solutions, there is one additional step that is required for the drug to be released. The drug should diffuse through such viscous media to reach the diffusion membrane which requires more time and delays the drug release to span 8 h instead of 2 h. Moreover, the special engineering of our ME is the primary reason for the sustained drug release for up to 24 h. As we mentioned before, ribavirin is located in the innermost aqueous layer of the ME. Therefore, to be released it has to pass through all three layers and cross two interfaces: the W/O interface which is located between the internal aqueous phase and the intermediate oil phase; and the O/W interface that is located between the intermediate oil phase and the external aqueous phase. Furthermore, ribavirin, as a BCS class III drug, is highly hydrophilic and its oil solubility is negligible (its n-octanol/water partition coefficient = −1.85) [61]. Therefore, the presence of the intermediate oil phase layer that surrounded its aqueous solution acts as a barrier that hinders its diffusion and greatly delays its release from the ME. After ribavirin passes through this oil phase obstacle, it should diffuse through the viscous external aqueous layer to reach the diffusion membrane and pass through it to the receiver chamber. All these steps are responsible for the sustained drug release and greatly prolonged the time required for ribavirin to be released from our ME. 

When comparing the drug release profiles of the two MEs, we determined that Carbopol 981 ME possessed a greater delayed drug release behavior than sodium alginate ME. This may be due to the difference in the polymer’s properties. Carbopol 981 is more viscous than sodium alginate at the concentrations used (Figure 2A). In addition, Carbopol 981 has a crosslinked structure, whereas the structure of sodium alginate is linear [60]. The higher viscosity and the crosslinking structure of Carbopol 981 result in a slower release rate because more time is needed for the drug to pass through this narrow polymeric network matrix. 

#### 3.3.6. In Vitro Transcorneal Permeability

**Figure 4:**
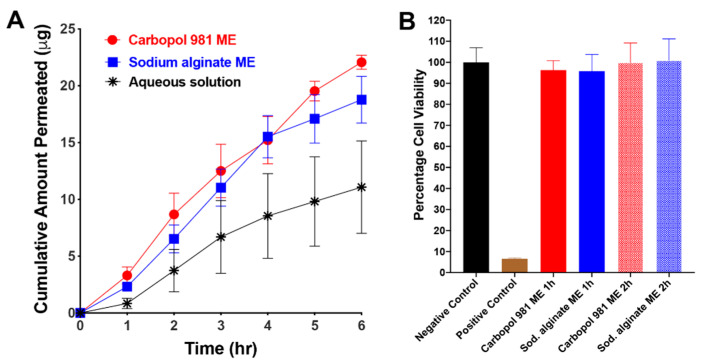
(**A**) In vitro transcorneal permeability profiles of ribavirin from Carbopol 981 and sodium alginate MEs and an aqueous solution as a control formulation (mean ± SEM, *n* = 4). (**B**) Histogram of the percentage cell viability of Carbopol 981 and sodium alginate MEs using HCLE cell line at different incubation times (mean ± SEM, *n* = 8).

The key point in a transcorneal permeability study is to maintain the vitality of the cornea through the entire experiment. To facilitate this, whole rabbit eyes were shipped overnight in Hanks balanced salt solution over wet ice, and during the experiment BSS-Plus solution was used as a permeability medium. BSS-Plus solution has a similar composition to aqueous humor and can maintain the vitality and integrity of the corneal tissue for longer time [62,63]. Figure 4A illustrates the corneal permeability profiles of Carbopol 981 ME, sodium alginate ME and the control aqueous solution of ribavirin. The results show that our ME greatly enhances the corneal permeability of ribavirin compared to the control formulation (*p* < 0.05), as indicated by the higher permeation rate, flux and permeability coefficients and a relative improvement of 2.71 folds relative to ribavirin aqueous solution (Table 3). This enhanced corneal permeability may be due to the presence of a high percentages of surfactants in the ME composition (Table 1). These surfactants can act as permeability enhancers and have the ability to improve the corneal permeabilities of poorly permeable drugs such as ribavirin through different mechanisms which may be transcellularly [64] or paracellularly [65,66]. Another cause for the enhanced corneal permeability of ribavirin may be the tiny droplet size of our ME that could help in improving the passive transport of ribavirin through cornea [55,56]. 

#### 3.3.7. In Vitro Evaluation of ME Effect on Cell Viability

An MTT assay was used to evaluate the effect of our ME on cell viability in vitro, in which the ME formulations were allowed to incubate with the cells for up to two hours. The cytotoxicity profiles of Carbopol 981 ME and sodium alginate ME after 1 h and 2 h of incubation are shown in Figure 4B. The figure demonstrates that our MEs are safe to the HCLE cell line at different incubation periods, as indicated by the high percentage of cell viability (≈100%) compared to the positive control (1% Triton X100, *p* < 0.0001). In contrast, no significant difference was found between the cell viability profile of our ME and the negative control (untreated cells, *p* > 0.05). Because this ME formulation is intended for topical treatment of an acute ocular condition and ribavirin is antiviral, a 15min incubation period for the ME with the HCLE cells would have been enough to judge its safety [5]. However, we extended the incubation period to 2 h to confirm the biocompatibility and safety of our ME formulations. 

### 3.4. In Vivo Safety and Ocular Tolerance Evaluation 

#### 3.4.1. Acute Ocular Toxicity Evaluation (Modified Draize Test)

**Figure 5:**
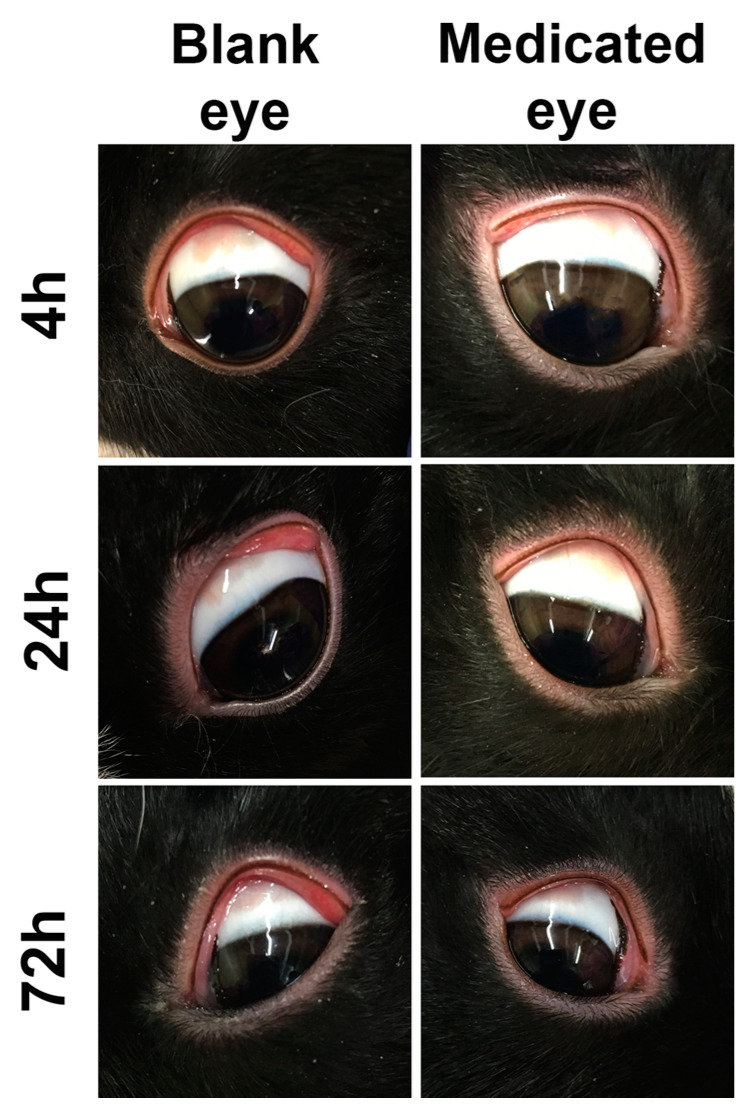
Photographs of Dutch belted rabbits’ eyes after 4 h, 24 h and 72 h of a single topical application of 100 µL blank or ribavirin-loaded Carbopol 981 MEs. There is no any sign of irritation, inflammation, toxicity or allergy.

The main purpose of the modified Draize test is to evaluate the formulation’s acute toxicity; such effects may happen after a single dose of some topical formulations intended to be used for treatment of acute diseases that require short-term treatments, such as infectious diseases. Carbopol 981 ME was selected for this experiment due to its promising in vitro results over the sodium alginate ME. Rabbits should remain in restrainers through the entire experiment (72 h) to prevent them from rubbing their eyes as an attempt to remove the bioadhesive formulations. Additionally, this rubbing action may result in some inflammation and irritation signs such as redness and swelling, which may interfere with the test results. Rabbits remained in their restrainer for the first 4 h, in accordance with the regulations of the Animal Care and Use review board of the University of Tennessee Health Science Center. Although there was no restraint after 4 h, we did not observe any inflammation or signs of irritation during examination after 4 h. Figure 5 illustrates the photographs taken of the rabbits’ eyes after 4 h, 24 h and 72 h post-application of both blank and medicated MEs. It is clear from the figure that all rabbits’ eyes, for blank and medicated formulations, were clear and did not show any signs of inflammation, irritation or allergic reactions. These results confirmed the safety of our ME for use in management of short-term eye conditions that result from acute ocular diseases. 

#### 3.4.2. Chronic Ocular Toxicity Evaluation

**Figure 6:**
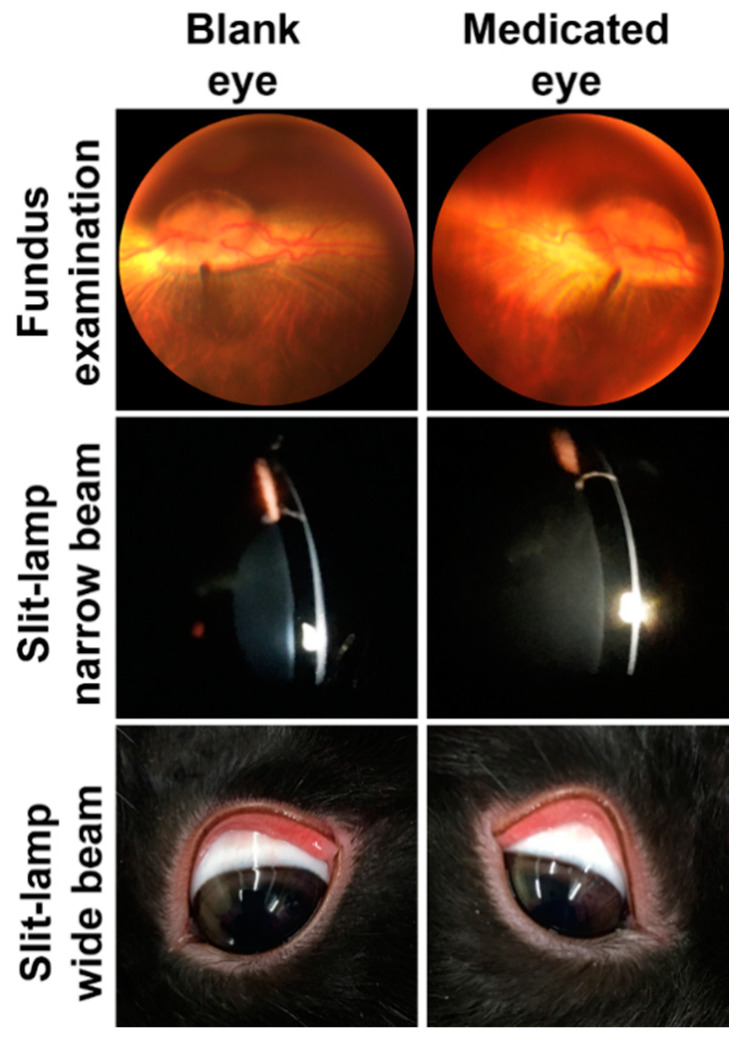
Photographs of fundus exam: narrow and wide beam slit-lamp biomicroscopic examination of Dutch belted rabbit eyes after 14 day of topical daily application of 100 µL of either blank or ribavirin-loaded Carbopol 981 ME. Fundus examination showed healthy retina and optic nerve discs in both eyes. Narrow beam slit-lamp biomicroscopic examination showed clear and smooth cornea, clear aqueous humor and normal lenses for both eyes. Wide beam slit-lamp biomicroscopic examination showed healthy eye surfaces that were free from any sign of irritation, inflammation, toxicity or allergy.

The main goal of this experiment was to evaluate the ocular toxicity of the ME upon daily dosing for extended period of time. It is useful to provide evidence of formulation safety when it is used for treatment of ocular diseases that may persist for a moderately longer period of time—infectious ocular diseases such as bacterial, viral and fungal eye infections. Figure 6 illustrates photographs of fundus examination, narrow and wide beam slit-lamp biomicroscopic examination of rabbits’ eyes after 14 days of daily dosing of 100 µL of either blank or ribavirin-loaded Carbopol 981 ME. The fundus examination photographs revealed healthy retinas and optic nerve discs with highly vascularized structure and normal blood supply. The narrow beam slit-lamp biomicroscopic examination of both eyes showed normal anterior chamber structures that were characterized by clear corneas with healthy epithelia and smooth surfaces free of any abrasion or swelling, clear aqueous humor that was free of cells or flare and a normal lens. The wide beam slit-lamp biomicroscopic examination revealed that the eye surfaces looked normal and did not show any signs of irritation, inflammation, toxicity or allergic reaction. 

## 4. Conclusions

The corneal pathway is the major pathway through which drug molecules penetrate the eyeball after topical application. Unfortunately, due to the special corneal structure, corneal drug penetration became a big challenge that faces drug delivery scientists. Recently, tweaking the composition of drug delivery systems greatly helped to improve the corneal drug penetration. Two important characteristics should be present in the topical ophthalmic formulation to achieve a better ocular bioavailability, including the presence of a penetration enhancer to enhance drug permeation through the tight junctions between the corneal epithelial cells or through the cell membranes, and the inclusion of bioadhesive agent to allow the formulation to remain in contact with corneal surface for enough time to help its penetration without rapid drainage. 

In the current study we succeeded in overcoming the limited drug corneal penetration by employing bioadhesive multiple ME technology. We used ribavirin, a poorly permeable BCS class III, as a model drug to demonstrate the ability of our lead ME formulation and improved its corneal permeability by 2.71-fold compared to ribavirin aqueous solution. To achieve our goal, we incorporated ribavirin in the internal aqueous phase of our multiple W/O/W ME, which is surrounded by an intermediate layer of oil phase, which plays the role of controlling the drug release from the innermost layer to the outermost layer to be ready for absorption. This special structure greatly prolongs the drug release for up to 24 h. The main feature that helped to improve ribavirin corneal permeability was the presence of several surfactants in our ME composition, which acted as penetration enhancers by both paracellular and intracellular mechanisms. Another factor in our ME that helped to improve the corneal penetration of ribavirin was the presence of a bioadhesive polymer in the external aqueous layer which maintained the ME on the corneal surface, thereby acting as a reservoir that continuously released the drug and prevented formulation drainage from the ocular surface. Finally, we concluded that our ME could work as a universal vehicle to deliver most water-soluble drugs to the eye with excellent ocular safety either for short or long-term use. 

## Figures and Tables

**Table 1:** Composition of ribavirin-loaded bioadhesive multiple W/O/W microemulsion (ME).

ME Ingredient (% *w*/*w*)	
Ribavirin	0.1
Labrafac Lipophile WL1349	3.96
Capryol 90	2.48
Soybean lecithin	2.48
Labrasol	4.5
Cremophor EL	4.5
Propylene glycol	18.0
Polymer	X
Water (to)	100

X: 0.4% sodium alginate in sodium alginate ME or 0.15% Carbopol 981 in Carbopol 981 ME.

**Table 2:** Droplet size, polydispersity index (PDI) and zeta potential of blank and medicated ribavirin ME formulations.

Type of ME	Mean Droplet Size (nm)		PDI		Zeta Potential (mV)	
	Blank	Medicated	Blank	Medicated	Blank	Medicated
Sodium alginate ME	10.8 ± 0.2	10.7 ± 0.02	0.304 ± 0.0	0.303 ± 0.0	−19.1 ± 0.56	−20.4 ± 1.1
Carbopol 981 ME	9.2 ± 0.04	9.1 ± 0.02	0.155 ± 0.0	0.163 ± 0.0	−23.7 ± 0.60	−21.4 ± 1.0

**Table 3:** In vitro transcorneal permeability parameters of ribavirin from Carbopol 981, sodium alginate MEs and an aqueous solution as a control.

Formulation	Rate of Permeation (dM/dt)	Flux (µg/cm^2^/min)	Permeability Coefficient (P) ×10^4^ (cm/min)	Relative Improvement
Aqueous solution	0.021 ± 0.005	0.033 ± 0.008	3.322 ± 0.778	1
Sodium alginate ME	0.027 ± 0.005	0.043 ± 0.007	4.276 ± 0.725	1.29
Carbopol 981 ME	0.057 ± 0.019	0.090 ± 0.030	8.991 ± 2.975	2.71
